# Repositioning renal denervation in resistant hypertension: evidence-based insights, stratified decision-making, and implementation pathways

**DOI:** 10.3389/fcvm.2026.1844520

**Published:** 2026-05-29

**Authors:** Min Li, Ding Yang, Xu Cheng, Qiong Wei, Dongmei Zhang

**Affiliations:** Key Laboratory of Chinese Internal Medicine of Ministry of Education and Beijing, Dongzhimen Hospital, Beijing University of Chinese Medicine, Beijing, China

**Keywords:** hypertension, renal denervation, renal sympathetic nerves, resistant hypertension, surgery

## Abstract

Renal denervation (RDN) is a catheter-based interventional approach that targets renal sympathetic nerves to modulate sympathetic overactivity, a key pathophysiological driver of resistant hypertension and associated cardiovascular complications. In contrast to pharmacological approaches, the therapeutic effect of RDN does not rely on patient adherence. Originally indicated for severe resistant hypertension, RDN has since shown consistent antihypertensive efficacy across a broad range of hypertensive patients in recent sham-controlled studies. In this review, we discuss the pathophysiological foundation underlying RDN, consolidate the current clinical evidence supporting its therapeutic efficacy, describe the spectrum of available ablation technologies, and evaluate its expanding applications in conditions driven by sympathetic overactivity.

## 1 Introduction

Hypertension is the leading modifiable risk factor for cardiovascular disease and all-cause mortality. Its global prevalence continues to rise due to population aging and increasing exposure to lifestyle-related risk factors (1). According to Global Burden of Disease (GBD) data, the burden of hypertension has increased substantially over the past three decades and has become one of the major contributors to disability-adjusted life years (DALYs) lost (2). In the United States, despite broad availability of antihypertensive medications and lifestyle interventions, only 23% of patients achieve blood pressure treatment goals, and blood pressure control rates appear to be worsening worldwide (3). More concerningly, approximately 10.3% of diagnosed hypertensive patients have resistant hypertension, defined as blood pressure ≥140/90 mmHg despite lifestyle optimization and treatment with ≥3 classes of antihypertensive medications—including a diuretic—at maximally tolerated doses, or as the need for ≥4 medications to reach target blood pressure (4–6).

Resistant hypertension carries a particularly heavy disease burden. Patients face markedly increased risks of cardiovascular events, target-organ damage, more frequent medical evaluations, and substantial economic burden (7, 8). However, distinguishing true resistant hypertension from pseudo-resistance is essential. Medication non-adherence is a major cause of pseudo-resistance, with global non-adherence rates reaching 27%–40%, especially in low- and middle-income countries and non-Western populations (9, 10). The pathophysiology of resistant hypertension is multifactorial, involving interactions among the renin-angiotensin-aldosterone system (RAAS), sympathetic nervous system (SNS), endothelin pathway, natriuretic peptides, and immune mechanisms (7). Among these, sympathetic overactivity plays a key role, forming the theoretical foundation for renal denervation (RDN) (11). Renal sympathetic nerves regulate renal blood flow, renin secretion, and tubular sodium reabsorption through afferent and efferent fibers, directly influencing central mechanisms of blood pressure control (12, 13).

Since the SYMPLICITY HTN-3 trial in 2014, renal denervation (RDN) has undergone validation and reassessment through multiple clinical trials. A new generation of studies, such as the SPYRAL HTN and RADIANCE series, has provided more robust evidence supporting its efficacy and safety. In November 2023, the U.S. Food and Drug Administration (FDA) approved two RDN systems—ReCor's Paradise ultrasound system and Medtronic's Symplicity Spyral radiofrequency system, marking a gradual transition of RDN technology into routine clinical practice (14).

A central challenge in the current RDN field is the substantial heterogeneity in individual treatment responses and the difficulty of predicting responders. Although population-level efficacy is well-established, individual responses vary widely, highlighting the need for precision stratification and personalized treatment strategies (15). Moreover, questions remain regarding long-term cardiovascular outcomes, cost-effectiveness compared with pharmacotherapy, and safety in patients with complex comorbidities (16). This review aims to comprehensively summarize the “repositioning” of RDN in resistant hypertension based on the latest evidence, analyze its mechanisms, clinical data, patient stratification strategies, and implementation pathways, and provide clinicians with evidence-based guidance to optimize clinical decision-making and improve patient outcomes.

## 2 Renal-sympathetic axis physiology and RDN mechanism of action

### 2.1 Anatomy and physiological function of renal sympathetic nerves

The renal sympathetic nervous system plays a central role in blood pressure regulation, serving as a critical link between central vascular control centers and renal function (17). Renal sympathetic nerves originate from the thoracolumbar spinal cord (T6–L2), extend via postganglionic sympathetic neurons, and ultimately innervate the adventitia of the renal arteries and renal parenchyma (18). Anatomical studies show that renal sympathetic nerve fibers are primarily located within the adventitia, at a depth of approximately 0.5–8.0 mm from the arterial lumen, with about 71.6% of fibers situated within 4 mm of the lumen (19).

**Figure 1:**
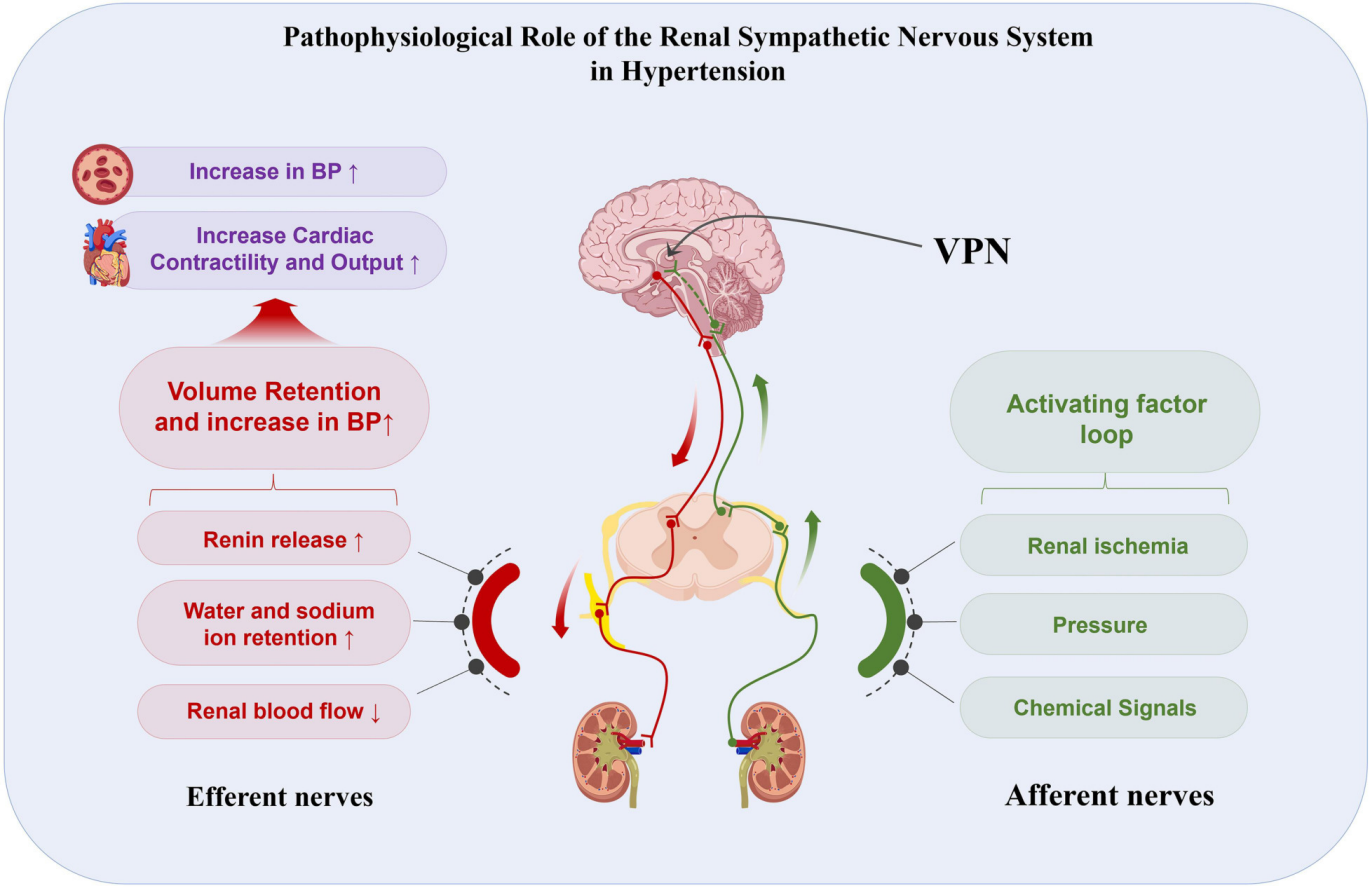
Pathophysiological role of the renal sympathetic nervous system in hypertension. Created using Biorender.

Renal sympathetic nerves are composed of efferent and afferent fibers that act synergistically in blood pressure regulation. Efferent fibers are predominantly noradrenergic and modulate renal blood flow, stimulate renin release, and enhance tubular sodium reabsorption through activation of *α*1-adrenergic receptors (20). Afferent fibers relay signals related to renal ischemia, pressure, and chemical changes back to the central nervous system, activating sympathetic outflow and generating a positive feedback loop (21) (Figure 1). Clinical studies have demonstrated markedly increased renal norepinephrine spillover in patients with resistant hypertension, indicating the critical pathophysiologic role of heightened renal sympathetic activity (22).

### 2.2 Device types of RDN

#### 2.2.1 Radiofrequency ablation technology

**Figure 2:**
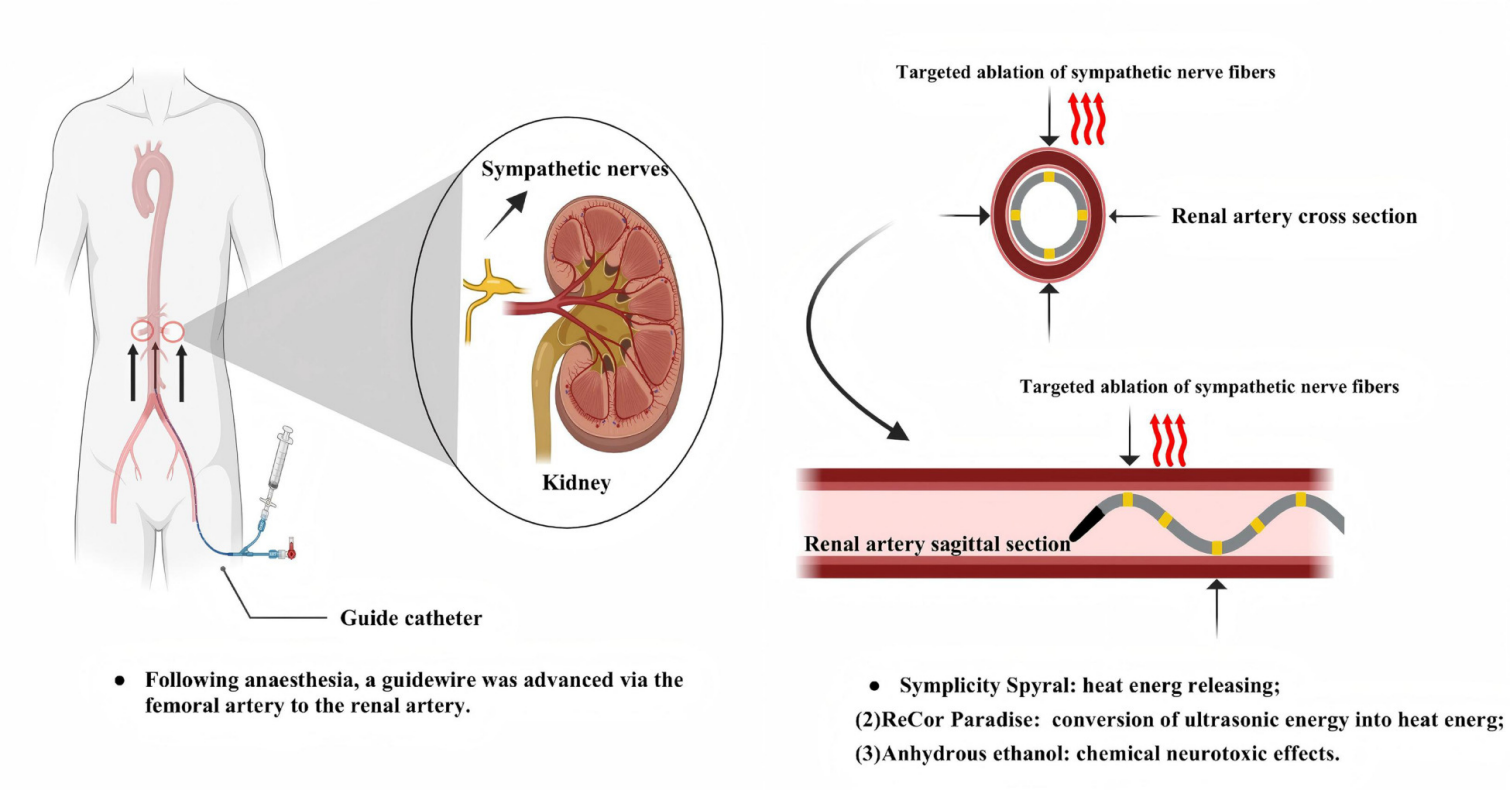
Schematic illustration of renal denervation techniques. Created using Biorender.

Radiofrequency RDN is the most widely used technique in current clinical practice. The representative device, the Medtronic Symplicity Spyral system, features a multi-electrode helical design that enables 360° circumferential ablation of both the main renal artery and its branches (23) (Figure 2). Radiofrequency energy denatures sympathetic fibers by heating tissue to 50–70°C, with each ablation lasting 120 s and power typically limited to <8 w for safety (24).

#### 2.2.2 Ultrasound ablation technology

Ultrasound RDN, represented by the ReCor Paradise system and FDA-approved in 2023, utilizes an intraballoon circumferential ultrasound transducer stabilized by a coolant-filled balloon, enabling noncontact circular ablation (25). The penetration depth of ultrasound energy reaches 7–8 mm, theoretically targeting deeper, more distant peri-arterial nerve fibers.

#### 2.2.3 Chemical ablation technology

Alcohol-mediated chemical neurolysis represents an emerging technique in which neurotoxic agents are injected into the perivascular region to ablate renal sympathetic nerves. This approach is simple and does not rely on thermal energy, but remains in early clinical development (26, 27).

### 2.3 Effects of renal denervation

RDN produces a durable and sustained antihypertensive effect. In the 36-month follow-up of the SPYRAL HTN-ON MED trial, patients receiving RDN maintained significantly greater blood pressure reductions than those in the sham group (28). In an animal study, renal nerve stimulation produced a pronounced elevation in blood pressure, which was significantly attenuated after selective ablation. Four weeks following the procedure, plasma levels of tyrosine hydroxylase and noradrenaline were decreased, and the magnitude of these decreases correlated with changes in systolic blood pressure (29). Emerging evidence suggests that RDN confers a spectrum of benefits beyond blood pressure reduction. In terms of cardiac effects, RDN can improve left ventricular hypertrophy and reduce arrhythmic burden (30–34). Among patients with refractory hypertension complicated by atrial fibrillation, RDN combined with pulmonary vein isolation significantly reduces atrial fibrillation recurrence compared to pulmonary vein isolation alone (35). Metabolically, RDN demonstrates potential benefits for hypertensive patients with diabetes by improving insulin sensitivity and glucose metabolism (36, 37). Regarding renal function, long-term follow-up indicates that RDN generally does not impair renal function, with some patients even showing mild improvement (38). These multi-dimensional physiological effects provide a solid mechanistic foundation for RDN's clinical positioning in managing refractory hypertension and its complications, while also suggesting its broad prospects in comprehensive cardiovascular management.

## 3 Key clinical evidence

### 3.1 Milestone achievement of the primary endpoint

The SPYRAL HTN-OFF MED Pivotal trial, the largest medication-withdrawal RDN study, enrolled 331 patients and, using a rigorous Bayesian statistical framework, confirmed the definitive efficacy of radiofrequency RDN (39). At 3 months, the primary endpoint of 24 h systolic BP reduction favored RDN, with a mean difference of −3.9 mmHg (95% BCI: −6.2 to −1.6) and a posterior probability of superiority exceeding 0.999. Office systolic BP reductions were more pronounced (−6.5 mmHg; 95% CI: −9.6 to −3.5). Although the absolute reduction in 24 h systolic blood pressure of −3.9 mmHg appears modest, the ability of RDN to achieve sustained blood pressure lowering covering the early morning, daytime, and nighttime periods has important clinical implications. A key strength of the OFF-MED trial lies in its exclusion of medication adherence as a confounding variable, allowing the observed between-group differences to be purely attributed to the RDN procedure itself—a finding of foundational importance for the establishment of the RDN evidence base. Notably, during the 3-month follow-up period, urinary screening revealed detectable antihypertensive medication in 9% of patients in the RDN group and 5% of those in the sham procedure group, suggesting that actual drug exposure may have led to an underestimation of the true treatment effect of RDN relative to sham. Furthermore, for ethical reasons, the OFF-MED trial required patients to resume antihypertensive pharmacotherapy after 3 months, and this short-term follow-up design may not have fully captured the complete blood pressure-lowering effect of RDN.

### 3.2 On-medication trial: the complexity and insights from SPYRAL HTN-ON MED

The results of the SPYRAL HTN-ON MED trial presented a more nuanced picture. At 6 months, although 24 h systolic BP decreased by 6.5 ± 10.7 mmHg in the RDN group and 4.5 ± 10.3 mmHg in the sham group, the between-group difference was only 1.9 mmHg (95% CI: −4.4 to 0.5; *P* = 0.12), failing to meet the prespecified primary endpoint (3). Despite not meeting the primary endpoint, office systolic BP decreased by 9.9 ± 13.9 mmHg, a reduction significantly greater than that of the sham group (*P* < 0.001). This finding is clinically meaningful because office BP remains the most widely used and clinically influential measure in hypertension management. Moreover, nighttime systolic BP reduction also favored RDN (−6.7 vs. −3.01 mmHg, *P* < 0.001). Part of the complexity observed in this trial may be attributed to the unforeseen influence of the COVID-19 pandemic. More than 80% of participants completed follow-up during the pandemic. Compared with those enrolled before the pandemic, baseline 24 h BP differed significantly, likely reflecting behavioral and lifestyle changes during lockdown periods. More importantly, patients in the sham group experienced a significantly higher intensification of antihypertensive therapy (3.5 vs. 2.9 drug classes at 6 months, *P* = 0.04). The sham control group enhanced blood pressure reduction by increasing antihypertensive medications, thereby diluting the true treatment effect of RDN. However, this precisely reflects the dilemma inherent in RDN clinical trials. From an ethical standpoint, allowing optimization of pharmacological therapy in the sham control group is necessary. Nevertheless, from the perspective of methodological rigor and scientific validity, such a design choice may bias the RDN effect toward a false-negative conclusion. Subsequently, the long-term follow-up results of the SPYRAL HTN-ON MED trial confirmed the efficacy of RDN. At 24 months, the RDN group experienced significantly greater mean reductions in ambulatory systolic BP (−12.1 ± 15.3 mm Hg vs. −7.0 ± 13.1 mm Hg; difference: −5.7 mmHg; *P* = 0.039) (40). At 36 months, 24 h SBP was controlled to <130 mmHg in 40% of RDN patients in the morning compared to 6% for the sham group (*P* = 0.021) and in 80% of the RDN patients at night compared to 39% in the sham group (*P* = 0.019) (41).

### 3.3 Clinical validation of ultrasound-guided RDN: the RADIANCE study

The RADIANCE-HTN SOLO trial demonstrated the efficacy of ultrasound-based RDN in patients with mild-to-moderate hypertension. A total of 146 participants underwent a 4-week medication washout and were randomized thereafter. At 2 months, daytime ambulatory systolic BP decreased 6.3 mmHg more in the RDN group than in the sham group (95% CI: −9.4 to −3.1; *P* < 0.0001). In the unblinded 12-month follow-up, patients in the RDN group maintained a sustained BP-lowering advantage and required fewer antihypertensive medications, confirming the durability of the treatment effect (42). The RADIANCE-HTN TRIO trial specifically evaluated patients with true resistant hypertension, all of whom were placed on a standardized triple fixed-dose combination therap. Among the 136 randomized patients, daytime ambulatory systolic BP at 2 months was reduced by an additional 4.5 mmHg in the RDN group compared with the sham group (95% CI: −8.5 to −0.3; *P* = 0.022) (43). This finding is particularly important as it demonstrates that RDN provides incremental BP reduction even in the most treatment-resistant patient population. As the pivotal study supporting U.S. FDA review, the RADIANCE II trial enrolled 224 patients with stage 2 hypertension (systolic BP 135–170 mmHg) who underwent a strict medication washout before randomizatio. At 2 months, daytime ambulatory systolic BP decreased 6.3 mmHg more in the RDN group than in the sham group (95% CI: −9.3 to −3.2; *P* < 0.001), meeting the primary endpoint (44). The success of this trial provided essential evidence for the FDA approval of the ReCor Paradise ultrasound renal denervation system. The key outcome measures of RDN clinical studies are summarized in Table 1.

**Table 1:** Summary of key outcome measures in clinical studies of renal sympathetic denervation.

Authors (Year)	Clinical study	Off/On-medicne	Device	Sample size (RDN/Sham)	Follow-up	Outcome measures	Mean between-group difference (95% CI) (mmHg)	*P* value (baseline adjusted)
Bhatt et al. (2014) (78)	SYMPLICITY HTN-3	On	Symplicity Flex	364/171	6 months	24 h SBP	−2.0 (−5.0, −1.1)	0.98
						Office SBP	−2.4 (−6.9, −2.1)	0.26
						Daytime SBP	−1.1 (−4.3, −2.2)	0.52
						Night-time SBP	−3.3 (−6.7, 0.1)	0.06
Kandzari et al. (2018) (79)	SPYRAL HTN-ON MED	On	Symplicity Spyral	38/42	6 months	24 h SBP	−7.4 (−12.5, −2.3)	0.0051
						Office SBP	−6.8 (−12.5, −1.1)	0.0205
Böhm et al. (39)	SPYRAL HTN-OFF MED	Off	Symplicity Spyral	166/165	3 months	24 h SBP	−4.0 (−6.2, −1.8)	0.0005
						Office SBP	−6.6 (−9.6, −3.5)	＜0.0001
Mahfoud et al. (28)	SPYRAL HTN-ON MED	On	Symplicity Spyral	38/42	24 months	24 h SBP	−11.2 (−18.4, −4.0)	0.0031
						Office SBP	−11.1 (−21.6, −0.5)	0.041
						Morning SBP	−11.2 (−21.7, −0.6)	0.039
						Daytime SBP	−10.2 (−18.0, −2.3)	0.013
						Night-time SBP	−12.9 (−21.1, −4.7)	0.0026
					36 months	24 h SBP	−10.0 (−16.6, −3.3)	0.0039
						Office SBP	−8.2 (−17.1, −0.8)	0.073
						Morning SBP	−11.0 (−19.8, −2.1)	0.016
						Daytime SBP	−8.9 (−16.5, −1.2)	0.024
						Night-time SBP	−11.8 (−19.0, −4.7)	0.0017
Kandzari et al. (3)	SPYRAL HTN-ON MED expansion	On	Symplicity Spyral	206/131	6 months	24 h SBP	−1.9 (−4.4, −0.5)	0.12
						Office SBP	−4.9 (−7.9, −1.9)	0.0015
						Daytime SBP	−1.2 (−3.8, −1.4)	0.37
						Night-time SBP	−3.7 (−6.5, −0.9)	0.0095
Azizi et al. (42)	RADIANCE-HTN SOLO	Off	Paradise	74/72	2 months	24 h SBP	−4.1 (−7.1, −1.2)	0.006
						Daytime SBP	−6.3 (−9.4, −3.1)	0.0001
Azizi et al. (43)	RADIANCE-HTN TRIO	On	Paradise	69/67	2 months	24 h SBP	−4.2 (−8.3, −0.3)	0.016
						Office SBP	−7.0 (−13.0, 0.0)	0.037
						Daytime SBP	−4.5 (−8.3, −0.3)	0.022
						Night-time SBP	−3.9 (−8.8, 1.0)	0.044
Kario et al. (2022) (80)	REQUIRE	On	Paradise	72/71	3 months	24 h SBP	−0.1 (−5.5, 5.3)	0.971
Azizi et al. (44)	RADIANCE II	Off	Paradise	150/74	2 months	24 h SBP	−6.2 (−9.1, −3.4)	＜0.001
						Office SBP	−5.4 (−9.0, −1.8)	0.004
						Daytime SBP	−6.3 (−9.3, −3.2)	＜0.001
						Night-time SBP	−5.8 (−9.0, −2.6)	＜0.001

### 3.4 Magnitude of treatment effect and clinical significance

The BP-lowering magnitude of RDN is comparable to that achieved with a single antihypertensive medication, typically ranging from 3 to 8 mmHg. Although this reduction may appear modest, it carries substantial cardiovascular protective benefits (45). Epidemiological data show that every 10 mmHg reduction in systolic BP lowers stroke risk by 27%, coronary heart disease risk by 17%, and all-cause mortality by 13% (46). According to the American Heart Association (AHA)/American College of Cardiology (ACC) Atherosclerotic Cardiovascular Disease (ASCVD) risk score, higher baseline cardiovascular risk correlates with greater estimated preventable cardiovascular events and more substantial blood pressure reduction. Consequently, patients with high cardiovascular risk may derive particular benefit from RDN treatment. At 3-year post-RDN follow-up, the mean 24 h systolic BP reduction was −8.9 ± 20.1 mm Hg in the overall cohort. For high-risk subgroups, systolic BP reductions were as follows: −10.4 ± 21.0 mm Hg in refractory hypertension, −8.7 ± 17.4 mm Hg in patients ≥65 years, −10.2 ± 17.9 mm Hg in diabetics, −8.6 ± 18.7 mm Hg in isolated systolic hypertension, −10.1 ± 20.3 mm Hg in chronic kidney disease, and −10.0 ± 19.1 mm Hg in atrial fibrillation (all *P* < 0.0001 vs. baseline) (47).

Among patients with varying baseline ASCVD risk scores, office and 24-hour blood pressure measurements at 6, 12, 24, and 36 months demonstrated similar sustained reductions maintained over 3 years. At the 3-year mark, patients with higher baseline cardiovascular risk exhibited higher rates of adverse events (40). These findings provide crucial guidance for clinical patient selection, facilitating personalized precision treatment approaches.

### 3.5 Safety evidence of RDN

Multiple large-scale randomized controlled trials have demonstrated a low incidence of acute adverse events following renal denervation (RDN) (Table 2). The most common adverse events are vascular access-site complications, such as hematoma and vascular injury. Serious safety events directly related to the procedure, including death, acute kidney injury, and renal artery perforation or rupture, are rare. Although current evidence indicates that RDN has a favorable overall safety profile with rare serious complications, this does not equate to zero risk. First, the available safety data are derived predominantly from published large-scale randomized controlled trials, which are subject to publication and selection biases. Second, the current follow-up duration is insufficient to adequately assess long-term risks. In real-world clinical practice, the safety of RDN depends not only on the technology itself but also on the operator's level of training, the systematic management capacity of the medical center, and the quality of risk communication in shared decision-making. In clinical practice, safety data should be regarded as the starting point, rather than the endpoint, of risk management, with the goal of minimizing potential risks during each procedure.

**Table 2:** Summary of safety events in clinical studies of renal rympathetic denervation.

Authors (Year)	Clinical study	follow-up	Incidence of safety events	
Bhatt et al. (2014) (78)	SYMPLICITY HTN-3	6 months	RDN (*n* = 364)	Death (2); Myocardial infarction (6); Increase in serum creatinine of >50% from baseline (5); Embolic event resulting in end-organ damage (1);Vascular complication requiring treatment (1); Hypertensive crisis or emergency (9); Stroke (4); Hospitalization for new-onset heart failure (9); Hospitalization for atrial fibrillation (5); New renal-artery stenosis of more than 70% (1)
			Sham (*n* = 171)	Death (1); Myocardial infarction (3); Increase in serum creatinine of >50% from baseline (1); Hypertensive crisis or emergency (9); Stroke (2); Hospitalization for new-onset heart failure (3); Hospitalization for atrial fibrillation (1)
Kandzari et al. (2018) (79)	SPYRAL HTN-ON MED	6 months	RDN (*n* = 38)	0^b^
			Sham (*n* = 42)	0^b^
Böhm et al. (39)	SPYRAL HTN-OFF MED Pivotal	3 months	RDN (*n* = 166)	^a^Hospitalization for hypertensive crisis/emergency (1)
			Sham (*n* = 165)	^a^New stroke (1)
Mahfoud et al. (28)	SPYRAL HTN-ON MED	36 months	RDN (*n* = 38)	Composite safety endpoint ^c^(1); New stroke (1); Hospitalisation for hypertensive crisis or emergenc (1)
			Sham (*n* = 42)	Composite safety endpoint ^c^(1); All-cause death (1)
Kandzari et al. (3)	SPYRAL HTN-ON MED expansion	6 months	RDN (*n* = 206)	Vascular complications (requiring surgical repair, intervention procedure thrombin, or blood transfusion) (2)
			Sham (*n* = 131)	Vascular complications (requiring surgical repair, intervention procedure thrombin, or blood transfusion) (1); New stroke (1)
Azizi et al. (42)	RADIANCE-HTN SOLO	2 months	RDN (*n* = 74)	Major adverse event ^d^ (0); Procedure-related pain lasting for >2 day (8); Need for renal artery angioplasty or stenting (1)
			Sham (*n* = 72)	Major adverse event (0); Procedure-related pain lasting for >2 day (8); Stroke, transient ischemic attack, cerebrovascular accident after 2 months (1)
Azizi et al. (43)	RADIANCE-HTN TRIO	2 months	RDN (*n* = 69)	Major access site complications requiring intervention (1); All-cause mortality (1); Acute myocardial infarction (1); Doubling of plasma creatinine (1); Procedure-related pain lasting for >2 days (12)
			Sham (*n* = 67)	Any coronary revascularisation (1); Doubling of plasma creatinine (1); Procedure-related pain lasting for >2 days (12)
Kario et al. (2022) (80)	REQUIRE	2 months	RDN (*n* = 72)	Vasospastic angina (Prinzmetal angina) (1); Puncture site hemorrhage (1); Cellulitis (1); Blood pressure decreased (1); Blood pressure increased (1); Postural dizziness (1)
			Sham (*n* = 71)	Pyrexia (1)
Azizi et al. (44)	RADIANCE II	1 month	RDN (*n* = 150)	0^e^
			Sham (*n* = 74)	0^e^

Data show number of patients with events.

a Safety events included: death, new-onset end stage renal disease, significant embolic event resulting in end-organ damage, Renal artery re-intervention, vascular complications, hospitalization for hypertensive crisis/emergency, new stroke, major bleeding (TIMI) and renal artery stenosis.

b Safety events included: death; new myocardial infarction, major bleeding, new onset end stage renal disease, serum creatinine elevation, significant embolic event resulting in end-organ damage, vascular complications, dissections perforations hospitalization for hypertensive crisis/emergency, new stroke and new renal artery stenosis (>70%).

c Defined as a composite of all-cause mortality, end-stage renal disease, embolic event resulting in end-organ damage, renal artery perforation requiring reintervention, renal artery dissection requiring reintervention, vascular complications, hospitalisation for hypertensive crisis or emergency, or new renal artery stenosis (>70%).

d Major adverse events included: death within 30 days, acute renal failure within 30 days, embolic event resulting in end-organ damage within 30 days, renal artery or other vascular complication requiring intervention within 30 days, hypertensive crisis within 30 days, new renal artery stenosis of more than 70% within 6 months.

e Safety events included: all-cause mortality, new onset end-stage renal disease (eGFR < 15 mL/min/m^2^ or need for renal replacement therapy), significant embolic event resulting in end-organ damage, renal artery perforation requiring an invasive intervention, renal artery dissection requiring an invasive intervention, major vascular complications requiring surgical repair, interventional procedure, thrombin injection, or blood transfusion, hospitalization for hypertensive or hypotensive crisis, Hospitalization for major cardiovascular-or hemodynamic- related events, new onset Stroke and new onset myocardial infarction in 30 days; New onset renal artery stenosis of more than 70% after 6 months, confirmed by CT or MR angiography.

## 4 When to choose RDN: core indications and clinical scenarios

As RDN moves from the research phase into clinical practice, it has become particularly important to establish a scientific and standardised patient stratification system and clinical decision-making pathways. The updates to the 2023 European Society of Hypertension (ESH) guidelines and the 2024 European Society of Cardiology (ESC) guidelines mark the formal establishment of RDN's status in the treatment of hypertension, shifting from the previous stance of “not recommended for routine use” to “may be considered as an adjunctive treatment option” (48). This shift provides clinicians with clear decision-making guidance, but simultaneously places higher demands on the precision of patient selection.

### 4.1 Definition of true resistant hypertension

True resistant hypertension is the primary indication for renal denervation (RDN), defined as persistently elevated blood pressure despite optimized drug therapy, both in-office and via out-of-office measurements. The updated ESH guideline emphasizes that diagnosis of true resistant hypertension must rely on out-of-office BP monitoring, significantly improving precision in patient selection.

### 4.2 Challenges of multi-drug intolerance and adherence

**Figure 3:**
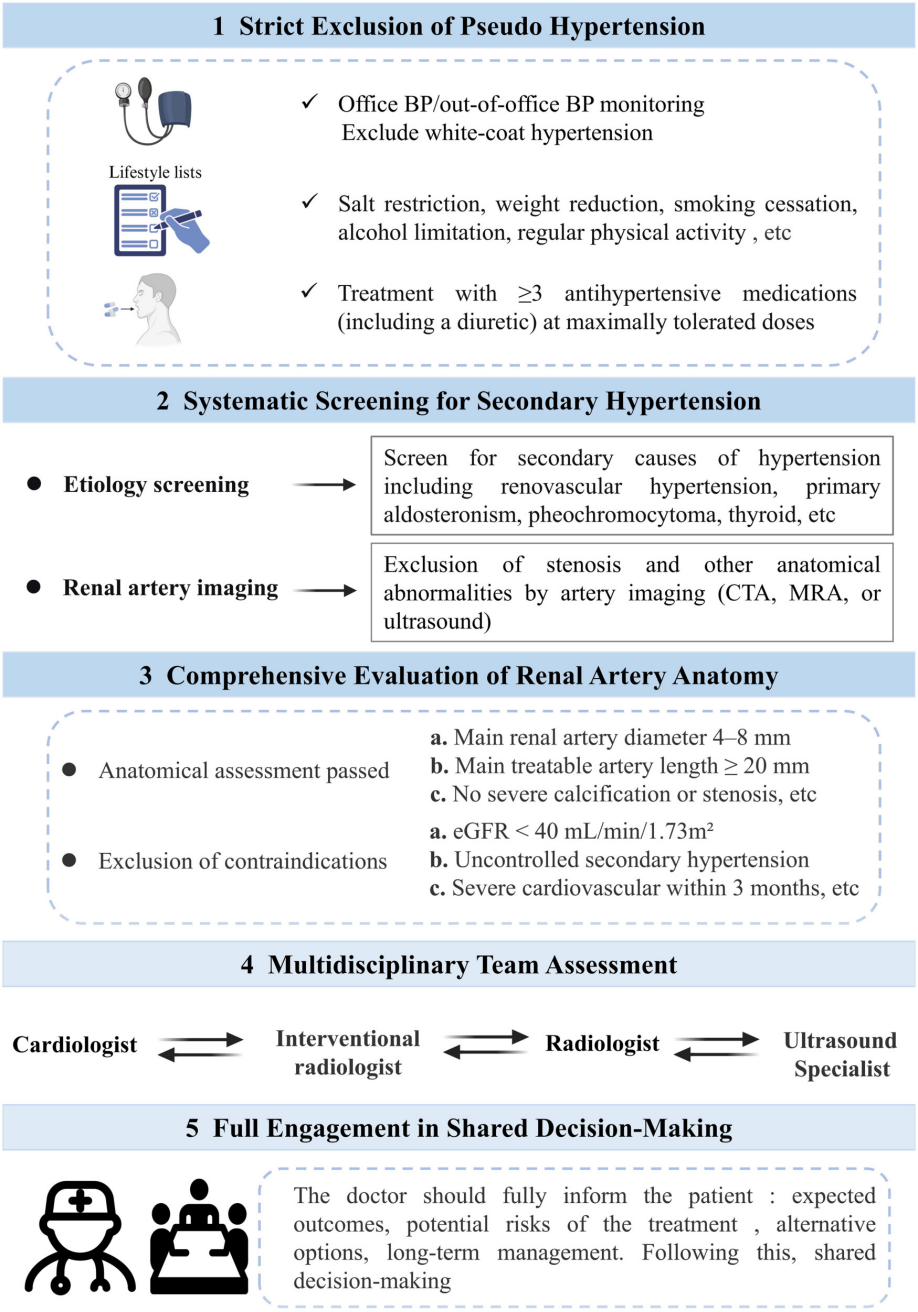
Clinical workflow for renal denervation candidate evaluation. Created using Biorender.

In real-world clinical practice, a considerable proportion of patients struggle to achieve adequate blood pressure control due to adverse drug reactions (such as cough or ankle edema) or the burden of polypharmacy, with some patients explicitly refusing further adjustment or intensification of their medication regimens (49). For these patients, renal denervation, as a one-off and adherence-independent interventional approach, offers distinct advantages. However, without a systematic and comprehensive evaluation, RDN may be prematurely viewed as a simpler option, thereby overlooking both the complex, reversible causes underlying resistant hypertension and the long-term risks and uncertainties associated with the procedure itself. Therefore, before considering RDN, clinicians should strictly adhere to a standardized diagnostic workup, which includes: excluding pseudo-resistant hypertension and white-coat hypertension through out-of-office blood pressure monitoring and drug concentration testing; systematically screening for secondary hypertension (such as primary aldosteronism or renal artery stenosis); and assessing whether the renal artery anatomy is suitable for the interventional procedure. It is only after thoroughly ruling out the above confounding factors and confirming the presence of true resistant hypertension with either intolerance or a suboptimal response to optimized medical therapy, that RDN may be regarded as a reasonable treatment option. This diagnostic process helps prevent RDN from being simplistically used as a clinical shortcut and ensures that its anticipated benefits are achieved in truly suitable patient populations. The screening process for RDN patients is shown in Figure 3.

### 4.3 Identification of special hypertension phenotypes

Certain hypertension phenotypes derive greater benefit from RDN and should be prioritized during patient selection: (1) Obstructive sleep apnea—related hypertension: Obstructive sleep apnea (OSA) induces sympathetic overactivation through intermittent hypoxia, leading to a blood pressure elevation mechanism centered on renal sympathetic nerve hyperactivity. Clinical evidence indicates that hypertensive patients with OSA exhibit more pronounced blood pressure reductions following renal denervation (RDN), particularly in nocturnal blood pressure (50, 51). However, current evidence is primarily derived from subgroup analyses, and dedicated randomized controlled trials specifically targeting the OSA population are still lacking. Moreover, the potential beneficial effects of RDN on OSA itself require further validation.In clinical practice, RDN may serve as an adjunctive treatment option for OSA patients who are intolerant or non—adherent to continuous positive airway pressure (CPAP) therapy, but it should not replace CPAP as the foundational treatment for OSA. (2) Salt-sensitive hypertension: Salt is one of the important environmental factors contributing to hypertension. In all types of hypertension, blood pressure increases with higher salt intake, albeit to varying degrees. Patients with salt-sensitive hypertension exhibit impaired renal sodium handling and markedly enhanced sympathetic nerve activity, making them an ideal therapeutic target for RDN. (3) Marked morning BP surge: Morning HBP is a strong predictor of future CAD and stroke events (52). RDN induces structural disruption of the renal sympathetic nerves, thereby avoiding the limitations associated with plasma half-life or medication adherence that are inherent to pharmacotherapy. During the morning blood pressure surge, when sympathetic nerve activity transitions from sleep-induced inhibition to activation, RDN reduces overall sympathetic tone in advance and attenuates the magnitude of the morning blood pressure rise. Multiple clinical trial data have further confirmed that RDN provides superior morning blood pressure control compared with pharmacological control (41, 53). However, it should be noted that the morning blood pressure surge is a multifactorial pathophysiological phenomenon involving sympathetic activation, the renin-angiotensin-aldosterone system, and cortisol secretion. RDN offers advantages specifically in controlling the sympathetic-dependent component of the morning surge.

### 4.4 Screening and evaluation of renal artery anatomy

Anatomical factors are critical in determining the feasibility of renal denervation (RDN). Standard anatomical screening typically involves bilateral renal artery angiography or CT angiography, with key evaluation criteria including: main renal artery diameter (generally required to be 3–8 mm), length (≥20 mm), presence of significant stenosis (>50%), degree of calcification, and the number and distribution of accessory renal arteries (54). Approximately 30% of patients have accessory renal arteries, and the strategy for managing them directly impacts the completeness of denervation. Available evidence indicates that treatment of accessory renal arteries is positively correlated with the magnitude of blood pressure reduction. Therefore, when technically feasible, denervation should be attempted on clinically significant accessory arteries (55).

### 4.5 Renal function assessment and safety thresholds

Renal function is a central parameter in the safety evaluation of RDN. The 2024 European Society of Cardiology guidelines indicate that RDN is not recommended for hypertensive patients with an estimated glomerular filtration rate (eGFR) of 40 mL/min/1.73 m² or lower (56). It is noteworthy that patients with chronic kidney disease (CKD) often exhibit heightened sympathetic nervous activity and may theoretically derive greater benefit from RDN. A recent analysis of the Global SYMPLICITY Registry DEFINE demonstrated that radiofrequency RDN provided clinically meaningful reductions in office and 24-hour ambulatory systolic blood pressure over three years in patients with CKD stage 3a (eGFR 45–60 mL/min/1.73 m^2^) and stage 3b (eGFR 30–45 mL/min/1.73 m^2^), without an increase in adverse events during the three-year period (57). In 2025, the Italian Society of Nephrology stated that in patients with uncontrolled or resistant hypertension and a significant reduction in glomerular filtration rate (<40 mL/min/1.73 m^2^), as well as in those on dialysis or living with a kidney transplant, the procedure significantly reduces blood pressure over time without negative effects on kidney function (58). For CKD patients whose medication options are limited due to impaired renal function, RDN offers a non-pharmacological option for blood pressure management. However, it should be noted that the three-year mortality rate in patients with CKD stage 3b reaches 15.2% (57), which is significantly higher than that in patients without CKD, reflecting the poor overall prognosis of this population. Although RDN can safely lower blood pressure, clinicians must engage in thorough risk communication and individualize decision-making, carefully weighing the benefits against the risks.

### 4.6 Technical considerations: radiofrequency vs. ultrasound

Radiofrequency (RF) and ultrasound (US) RDN represent the two most evidence-based technologies in the current RDN landscape, differing in their mechanisms of action, ablation strategies, efficacy profiles, and safety outcomes (Tables 3, 4). The RADIOSOUND-HTN trial is the only prospective, randomized controlled study to date that directly compares these two technologies. At three months post-procedure, the US group demonstrated a significantly greater reduction in blood pressure compared to the main renal artery RF ablation group (–13.2 ± 13.7 vs. −6.5 ± 10.3 mmHg; mean difference −6.7 mmHg; *P* = 0.043), whereas no significant difference was observed between the two RF subgroups (main artery only vs. main plus branch ablation) (25). At six months, the reduction in systolic ambulatory blood pressure monitoring (ABPM) from baseline differed significantly among the treatment arms (*P* = 0.017 for between-group comparison). However, no significant difference in ABPM values was found between the US-RDN and RF-RDN groups at 12 months (59). Furthermore, at the 2024 annual meeting of the German Society of Cardiology, Fengler K et al. reported that the 24-month follow-up results showed no significant intergroup differences in blood pressure.

**Table 3:** Comparison of device characteristics and procedural parameters between radiofrequency and ultrasound renal denervation.

Comparison dimension	Radiofrequency ablation	Ultrasound ablation
Representative Device	Symplicity Spyral™ (Medtronic)	Paradise™ (ReCor Medical)
Energy Type	High-frequency current, thermal coagulation necrosis	Focused ultrasound beam, thermal and mechanical effects
Targeted selectivity	selectivity	non-selectivity
Depth of energy penetration	Approximately 3–4 mm	Approximately 6–7 mm
Ablation Strategy	Point-by-point ablation, covering all four quadrants	Balloon-centered transducer, circumferential continuous ablation
Catheter Size	6Fr (smaller), Femoral access	7Fr, Femoral access
Ablation Duration	Simultaneous ablation at multiple points, approximately 45–60 s per ablation	Approximately 7 s per ablation
Ablation Range	Can access branch arteries (≥3 mm)	Primarily limited to the main renal artery

**Table 4:** A comparison of the blood pressure-lowering effects of different RDN techniques in patients with refractory hypertension: the RADIOSOUND-HTN trial.

Authors (Year)	Follow-up time	Sample size	Outcome measures	Reduction in BP			RFM-RDN VS USM-RDN	RFB-RDN VS USM-RDN	RFM-RDN VS RFB-RDN	baseline-adjusted *P* value
				RFM-RDNa	RFB-RDNb	USM-RDNc				
Fengler Ki et al. (25)	3 months	*N* = 120 (38:37:42)	Daytime SBP	−6.5 ± 10.3	−8.3 ± 11.7	−13.2 ± 13.7	*P* *=* 0.043	*P* *=* 0.22	*P* > 0.99	*P* = 0.038
			Daytime DBP	–	–	–	*P* *=* 0.025	*P*＞0.05	*P* > 0.05	*P* = 0.025
			Nighttime SBP	−2.1 ± 13.3	−5.1 ± 16.0	−10.2 ± 13.9	*–*	*–*	*–*	*P* = 0.32
			24 h SBP	–	–	–	*P* *=* 0.029	*P* > 0.05	*P* > 0.05	*P* = 0.027
			24 h DBP	–	–	–	*P* *=* 0.015	*P* > 0.05	*P* > 0.05	*P* = 0.018
Fengler Ki et al. (59)	6 months	*N* = 102	24 h SBP	−6.0 ± 11.0	−4.8 ± 12.1	−12.1 ± 11.5	*P* > 0.05	*P* > 0.05	*P* < 0.05	*P* *=* 0.017
			24 h DBP	–	–	–	*P* > 0.05	*P* > 0.05	*P* > 0.05	*P* > 0.05
			Daytime SBP	−6.1 ± 12.5	−5.0 ± 12.2	−13.0 ± 12.3	–	–	–	*P* *=* 0.015
			Daytime DBP	–	–	–	–	–	–	*P* > 0.05
	12 months	*N* = 83	24 h SBP	–	–	–	*P* > 0.05	*P* > 0.05	*P* > 0.05	*P* > 0.05
			24 h DBP	–	–	–	*P* > 0.05	*P* > 0.05	*P* > 0.05	*P* > 0.05
			Daytime SBP	–	–	–	–	–	–	*P* > 0.05
			Daytime DBP	–	–	–	–	–	–	*P* > 0.05
	24 months	*N* = 70	Daytime SBP	–	–	–	–	–	–	*P* > 0.05
			Daytime DBP	–	–	–	–	–	–	*P* > 0.05

Abbreviations: RFB-RDN, radiofrequency main renal artery ablation with an additional side branch ablation; RFM-RDN, radiofrequency main renal artery ablation; USM-RDN, ultrasound ablation of the main renal arteries.

Anatomical studies have confirmed that partial proportion of these fibers lie more than 3–4 mm from the luminal surface (60). From a mechanistic perspective, the effective thermal lesion radius of RF ablation is typically limited to 3–4 mm around the catheter contact point, posing a risk of incomplete ablation of deeper nerve fibers. In contrast, US ablation offers a penetration depth of 6–7 mm and employs a circumferential ablation pattern, theoretically enabling more complete ablation of sympathetic nerves located farther from the vessel wall. It is important to note that during the 3- and 6-month follow-up periods, patients in all groups maintained stable antihypertensive medication regimens. Consequently, the observed intergroup differences during this window can be relatively purely attributed to the distinct effects of the RDN technologies themselves. The subsequent optimization of antihypertensive drug therapy after six months may have masked the differential efficacy between the techniques. Additionally, the loss to follow-up increased from 120 patients at baseline to only 83 at 24 months (a dropout rate of approximately 42%), which compromises the statistical power for long-term comparisons. Regarding safety, multiple randomized controlled trials have consistently demonstrated that the incidence of major adverse events associated with either RF- or US-based RDN is not statistically different from that of sham-controlled procedures, nor is there a significant difference in safety profiles between the two technologies (61). In summary, the two technologies have distinct emphases. Ultrasound-based main-artery ablation appears to offer superior short-term, device-specific efficacy, making it potentially more suitable for patients with favorable anatomy in whom branch ablation is to be minimized. Radiofrequency ablation, in contrast, has a broader evidence base and greater device accessibility. In clinical practice, the choice of technology should transcend a simplistic dichotomy and instead move toward individualized decision-making that integrates patient-specific anatomical characteristics, patient preferences, center experience, and health economic factors. Future large-scale, head-to-head randomized trials with extended follow-up are warranted to delineate the optimal indications for each technology across different patient subgroups, thereby providing a more robust evidence base for precision RDN therapy.

### 4.7 Complementarity and integration with other device-based strategies

In addition to RDN, other device-based strategies such as baroreflex activation therapy (BAT) and carotid body denervation (CBD) also modulate sympathetic nerve activity through distinct mechanisms, theoretically offering potential complementarity to RDN (62, 63). However, it must be emphasized that, compared with the substantial randomized controlled trial evidence available for RDN, the clinical evidence for BAT and CBD primarily derives from small-sample studies or early-stage exploratory trials, and their long-term safety and efficacy require further validation. Therefore, in current clinical practice, RDN remains the device-based therapeutic option with the most robust evidence.

### 4.8 Standardized clinical decision pathway for RDN

#### 4.8.1 Multidisciplinary team model

The successful implementation of renal denervation (RDN) requires the establishment of a standardized multidisciplinary collaborative model. The core team should include hypertension specialists (responsible for patient selection and long-term management), interventional cardiologists (responsible for procedure performance), radiologists (responsible for anatomical assessment), and clinical pharmacists (responsible for medication optimization) (64). The key to effective collaboration lies in establishing standardized referral criteria, evaluation protocols, and follow-up pathways.

#### 4.8.2 Framework for shared decision-making

Shared decision-making with active patient involvement is a crucial component of RDN clinical practice. Physicians must thoroughly explain to patients the expected treatment effect (typically a 5–8 mmHg reduction in blood pressure), potential risks, alternative treatment options, and the necessity of long-term management (65). It is important to clearly communicate that RDN is not a “cure” for hypertension, but rather an integral component of a comprehensive management strategy. The decision-making process should carefully consider the patient's values, lifestyle preferences, and risk tolerance.

#### 4.8.3 Post-procedural follow-up and efficacy assessment

A standardized follow-up protocol is essential for the long-term success of RDN. Recommended follow-up time points include 1 week (for safety assessment), 1, 3, 6, and 12 months (for efficacy assessment), and annually thereafter (66). Efficacy assessment should prioritize out-of-office blood pressure monitoring, primarily using 24 h ambulatory blood pressure monitoring or properly conducted home blood pressure monitoring. Any medication adjustments should be made gradually, avoiding premature or excessive reduction.

With accumulating clinical experience and continuous technological refinement, patient selection strategies for RDN are expected to become more precise and individualized. Future directions include biomarker-guided patient selection, artificial intelligence-assisted decision support systems, and genotype-based personalized treatment plans. These advances are poised to further enhance the clinical effectiveness of RDN, allowing a greater number of patients with resistant hypertension to benefit.

## 5 Health economics and accessibility

### 5.1 Positive signals from economic value assessment

In addition to evidence of clinical efficacy, safety, and clinical effectiveness, healthcare payers increasingly require cost-effectiveness analyses to judge the value of health technologies. As the first minimally invasive interventional procedure for hypertension, is RDN truly effective and affordable from an economic perspective? A recent cost-effectiveness analysis based on the U.S. healthcare system showed that the incremental cost-effectiveness ratio (ICER) for radiofrequency RDN was $32,732 per quality-adjusted life year (QALY), well below the $50,000 threshold for a high-value treatment (67). An analysis based on the RADIANCE-HTN TRIO trial, conducted within the UK healthcare system, demonstrated that ultrasound RDN is cost-effective in patients with resistant hypertension (68). When discussing cost-effectiveness, the following limitations should be noted to avoid overly optimistic interpretation: (1) Long-term outcome data remain insufficient: the long-term effects of RDN on cardiovascular endpoints (e.g., myocardial infarction, stroke, death) still require validation with follow-up exceeding ten years; (2) Cost-effectiveness analyses are highly dependent on model assumptions: variations in parameters such as discount rates, time horizons, and complication rates can lead to substantially different conclusions; (3) Heterogeneity in patient response to RDN: in patients who show no significant blood pressure reduction after the procedure, the cost-effectiveness will be greatly reduced; (4) The future decline in device prices is uncertain, which will directly affect projections from long-term cost-effectiveness models. In summary, when interpreting the cost-effectiveness of RDN, the above limitations should be considered carefully, and extrapolation to all patient populations or all healthcare settings should be avoided.

### 5.2 Market prospects and cccess challenges

The global renal denervation device market is experiencing rapid growth. A market research reports indicate that the RDN market is projected to grow from USD 340.4 million in 2023 to USD 3.62 billion by 2030, representing a compound annual growth rate of 40.2%. Europe leads the market with a 45.6% share, largely attributable to the high prevalence of hypertension and well-established healthcare infrastructure. Increased healthcare investment in countries such as China, Japan, and India is driving market expansion. Nevertheless, the widespread adoption of RDN still faces significant access challenges. First, the high initial device cost constitutes a major barrier in resource-limited healthcare settings. Second, technical requirements demand specialized centers with interventional capabilities and experienced operators, which limits dissemination in primary care institutions. Third, the lag in policy adoption by various national payers affects real patient affordability.

### 5.3 Integration into value-based healthcare models

The unique value of RDN lies in its single-intervention, long-term benefit profile, which contrasts sharply with the traditional chronic disease medication management model (Table 5). Economic analyses indicate that, although the upfront cost of RDN is high, it is a high-value and cost-effective intervention by reducing long-term medication expenses, lowering cardiovascular event rates, and decreasing healthcare resource utilization (69). This value proposition is particularly suited for healthcare systems with constrained budgets and a focus on long-term cost control. Furthermore, the integration of smart blood pressure monitoring devices, artificial intelligence -assisted risk prediction, and individualized treatment adjustment can maximize the clinical benefit of RDN. In the early post-procedure phase, smart monitoring and artificial intelligence can help confirm procedural efficacy and identify poor responders. During the stable phase, follow-up can be stepped down to low intensity (70). This integrated model is expected to become an important direction for precision management of hypertension in the future.

**Table 5:** Comparison of cost characteristics between medication management and renal denervation models.

Cost dimension	Medication management model	RDN model
Cost Curve	Linear continuous accumulation (lifelong)	Early single high peak, then flattens out
Main Cost Components	Medication costs, regular laboratory tests, outpatient visits	Procedural consumables, equipment, operator fees
Adherence-Related Costs	Missed doses/discontinuation lead to uncontrolled BP, increasing hospitalization costs for complications	Independent of adherence, avoiding adherence-related costs
Management Costs	Persistently present	Significantly reduced (annual follow-up only)

## 6 Controversies and evidence gaps in RDN

### 6.1 The challenge of predicting individual response heterogeneity

One of the key controversies in the field of RDN lies in the marked heterogeneity of individual responses to treatment. How to pre-identify potential non-responders remains an unsolved problem due to the lack of reliable and broadly applicable predictive models. Although multiple candidate predictors have been investigated, only baseline blood pressure level has been consistently validated as a relatively reliable positive predictor to date, whereas the predictive value of factors such as heart rate, pulse wave velocity, and arterial stiffness remains highly controversial (71). Whole-exome sequencing studies have failed to identify genetic variants significantly associated with RDN efficacy, suggesting that genetic testing currently has limited utility in guiding patient selection (72). Regarding biomarkers, although certain molecules such as mid-regional pro-adrenomedullin have shown some association with treatment response, their clinical predictive performance still requires validation through large-scale, prospective studies (73). Overall, the prediction of RDN efficacy remains in its early exploratory stage, with most existing predictors derived from retrospective analyses or small-sample studies, lacking rigorous external validation from large-scale prospective evidence. Therefore, the development of machine learning-based predictive models integrating multidimensional data (including clinical characteristics, imaging parameters, biomarkers, intraoperative electrophysiological responses, etc.) may represent an important direction toward overcoming this bottleneck.

### 6.2 Technical limitations affecting the successful validation of RDN

Another major controversy currently facing RDN is the lack of real-time, reliable methods for confirming procedural success. Unlike other interventional procedures, This dilemma not only leads to difficulties in technical standardization and heterogeneity of outcomes, but also becomes a core bottleneck restricting the transition of RDN from empirical practice to evidence-based precision therapy. At present, relevant clinical research is advancing steadily, with initial progress achieved in the mapping/selective renal denervation (msRDN) system. Chinese investigators have used intraoperative electrical stimulation mapping to identify hot spots that truly require intervention, defined as sites where electrical stimulation induces a sustained rise in systolic blood pressure exceeding 5 mmHg. Following ablation, electrical stimulation can be reapplied immediately to verify the ablation effect (by observing whether blood pressure declines). msRDN is currently the only technique that has achieved immediate post-procedural verification capability in humans and has been successfully implemented in several centers in China, albeit with a limited number of cases to date. In terms of imaging validation, intravascular ultrasound (IVUS) and optical frequency domain imaging can be used to assess the extent of ablation injury immediately after RDN (74). however, current evidence is derived exclusively from animal experiments. Additionally, some investigators have employed a time-varying two-element Windkessel model to analyze the relationship between renal artery pressure and blood flow. In rabbit and porcine RDN models, this technique can detect a significant reduction in negative admittance gain and negative phase shift within the characteristic frequency range (0.2–0.75 Hz) following denervation, whereas conventional hemodynamic parameters (e.g., mean renal blood flow, mean renal vascular resistance) show no significant changes (75). It should be noted that this technique remains at the experimental stage and has not yet entered clinical practice. Potential directions for overcoming the current technological validation bottleneck include: (1) developing more reliable non-invasive or minimally invasive methods for assessing neural function; (2) establishing multimodal intraprocedural endpoint metrics that integrate hemodynamic, neurophysiological, and imaging parameters; (3) conducting multicenter randomized controlled trials with unified designs and clearly defined endpoints to systematically quantify the relationship between technical success and clinical success.

### 6.3 Lack of long-term clinical outcome evidencence

Although the evidence supporting the blood pressure-lowering effect of RDN is increasingly robust, direct evidence of its impact on hard clinical endpoints (e.g., cardiovascular events, mortality) remains lacking. Existing studies have primarily estimated cardiovascular benefits based on blood pressure changes, but the reliability has not been directly validated (76, 77). Designing large-scale randomized controlled trials specifically powered for cardiovascular endpoints faces multiple challenges, including the need for a very large sample size, long follow-up duration, high costs, ethical concerns, and confounding due to medication cross-over and adjustments. Whether blood pressure reduction following RDN translates into cardiovascular risk reduction comparable to that achieved with pharmacological therapy remains unclear. In the short term, real-world evidence and registry-based studies, using methods such as propensity score matching or target trial emulation, can provide complementary evidence. In the long term, large-scale, long-follow-up cardiovascular outcome trials remain the gold-standard approach to establish the clinical value of RDN. Based on the current state of evidence, the clinical application of RDN should adhere to the following principles: (1) select appropriate candidates based on rigorous multidimensional assessment to avoid indiscriminate adoption; (2) implement transparent informed consent, explicitly informing patients that the blood pressure-lowering effect is well established, but cardiovascular benefit remains to be validated; (3) establish long-term post-procedural follow-up mechanisms, enroll patients in registries, and continuously accumulate real-world evidence.

## 7 Future directions

As clinical experience accumulates and techniques continue to improve, patient selection strategies for RDN will become increasingly precise and personalised. Future developments will include biomarker-guided patient selection, AI-assisted decision support systems, and genotype-based personalised treatment plans. These advances will further enhance the clinical efficacy of RDN, enabling more patients with resistant hypertension to benefit from the treatment.

## 8 Literature search strategy

To ensure transparency and reproducibility, we conducted a structured literature search. We retrieved English-language publications published up to December 2025 from the PubMed, EMBASE, and Cochrane Library databases, using search terms including “renal denervation”, “resistant hypertension”, “multicenter”, “randomized controlled trial”, and combinations thereof. Randomized controlled trials with duplicate enrollments or sample sizes less than 50 cases were excluded. Ultimately, 10 multicenter randomized controlled clinical trials were included.
